# Supplementary material for: Transient exposure to oxygen or nitrate reveals ecophysiology of fermentative and sulfate‐reducing benthic microbial populations
Source: Environ Microbiol. 2017 Sep 15;19(12):4866–81. doi: 10.1111/1462-2920.13895 (PMC5763382; doi:10.1111/1462-2920.13895)
Supplement: Supplementary file 1 — Fig. S1. Visualization of assembled contigs of the six metagenomes (Oxy‐1, Oxy‐2, Nit‐1, Nit‐2, Con‐1, Con‐2) after binning. Bins A‐R are indicated with capital letters. Letters in parentheses show large bins that were detected, but had assembled better in another sample. Refer to this graph to link the metatranscriptomic contig identifier (Dataset 1) to the metagenome that contains the respective bin/contig. Assembled contigs are archived in the SRA under: Oxy‐1: LQAE00000000; Oxy‐2:LQAF00000000; Nit‐1: LQAG00000000; Nit‐2: LQAH00000000; Con‐1: LQAI00000000; Con‐2: LQAJ00000000 Fig. S2. The optical density and dilution rate remained constant during the entire experiment. This means that microbial growth and biomass remained constant in each culture and were not affected by the different treatments. Based on the dilution rate /growth rate of 0.36 – 0.4, the organisms had an average doubling time of 40–45 h. Optical density was measured as absorbance at 600 nm wavelength (OD600). Fig. S3. The concentrations of sulfide, oxygen or nitrate during one treatment cycle. The sulfide concentration decreased during aeration and recovered when the aeration was terminated after 30 min (A). The sulfide concentration in the nitrate treatment (B) was fluctuating, but constant. Oxygen reached a concentration of 1.4% saturation during the treatment (C) and was flushed out of the culture after 30 min using argon. Nitrate reached 0.5 mM during the treatment and was metabolized by the microorganisms within 100 min (D). The sulfur concentration in the oxygen treated cultures was high immediately after the aeration and then decreased (E), whereas in the nitrate‐treated cultures seemed to peak at around 100 min after the treatment. Fig. S4. Principal component analysis (Edge‐PCA) of the six Janssand continuous culture metagenomes based on relative abundance of 37 core marker gene sequences as detected by Phylosift. Fig. S5. Relative transcriptional activity for key genes involved in sulfa [file EMI-19-4866-s001.docx]

**Supporting Information**

**For article**

**“Transient exposure to oxygen or nitrate**

**reveals ecophysiology of fermentative and sulfate-reducing benthic microbial populations”**

Zainab Abdulrahman Beiruti^1^, Srijak Bhatnagar^2^, Halina E. Tegetmeyer^1,3^,

Jeanine S. Geelhoed^1,4^, Marc Strous^1, 3, 5^, S. Emil Ruff^1, 5, †^

File contains:

Supporting Experimental Procedures

Supporting Results

Supporting Figures S1 – S9

Supporting Tables S1, S2

**Supporting Experimental Procedures**

Continuous culture setup

After inoculation, oxygen was removed from the culture headspace (0.1 l) by flushing with Argon (10 ml/min) for two days with a mass flow controller (Alicat Scientific). During the first month of cultivation, the cultures were operated in repeated fed-batch mode. Fresh medium was supplied at a rate of 0.17 l day^-1^. Once per three days, medium was removed from the culture while purging Argon (10 ml min^-1^) into the culture. To maintain anoxic conditions, purging with Argon was continued for 1 h after medium removal. After one month and onward, the culture was operated as a chemostat with continuous removal of spent medium via an overflow.

Organic carbon mixture, trace element solution and pH buffer

The organic carbon mixture consisted of 1.1 mM D-glucose, 1.7 mM acetic acid, and a mixture of amino acids (in mM: L-glutamic acid 0.38, L-aspartic acid 0.65, L-alanine 0.85, L-serine 0.46, L-tyrosine 0.099, L-histidine 0.035, L-methionine 0.088). In addition, 0.2 mM Na-phosphate, 17 μM FeSO_4_, 0.2 ml l^-1^ trace element solution (in mg l^-1^: ZnCl_2_ 69, MnCl_2_×4 H_2_O 100, H_3_BO_3_ 60, CoCl_2_×6 H_2_O 120, CuCl_2_×2 H_2_O 10, NiCl_2_×6 H_2_O 25, Na_2_MoO_4_×2 H_2_O 25, AlCl_3_×6 H_2_O 25, in 0.1% HCl) and 0.2 ml l^-1^ Se/W solution (in mg l^-1^: Na_2_SeO_3_×H_2_O 6, Na_2_WO_4_×2 H_2_O 8, in 0.04% NaOH) were added. The medium also contained 20 mM of 4-(2-hydroxyethyl)piperazine-1-ethanesulfonic acid (HEPES) to buffer the pH of the culture.

RNA extraction

RNA was extracted from a 2 ml sample of all six cultures on day 311 (oxygen treated cultures), on day 327 (nitrate treated cultures) and on day 300 (untreated control cultures), as previously described (Hanke *et al.*, 2016). For those cultures with cyclic oxygen or nitrate supply, RNA was extracted an hour before the treatment and immediately after the treatment subsided, i.e. when oxygen and nitrate concentrations had decreased to background values, 30 min and 240 min after the treatment commenced, respectively (Fig. S3C, D). . Between sampling and extraction, the samples were stored at -20ºC in RNA stabilization solution (RNA*later*®, Invitrogen). Extraction was performed by adding 1 ml of TRI Reagent^®^ Solution (Ambion), bead beating in 2 ml vials filled with 300 µl of glass beads, diameter 0.1 mm, for 45 s at 6.5 m s^-1^, incubation at room temperature (RT) for 5 min and centrifugation at 4500 × *g* at 4°C. The supernatant was transferred to new vials, 200 µl chloroform were added, followed by vigorous shaking for 15-30 s, incubation at RT for 10 min, and centrifugation at 12,000 × *g* for 15 min at 4°C. The upper aqueous phase was collected and RNA was precipitated on ice for 20 min after adding 500 µl of isopropanol. After centrifugation at 20,000 × *g* for 25 min at 4°C, the RNA pellets were washed three times with 75% ice cold ethanol, air dried for 15 min, and resuspended in sterile TE buffer (pH 8.0). The extracted RNA was treated with DNase (Promega, Mannheim, Germany) and purified with Rneasy MinElute spin columns (Qiagen, Düsseldorf, Germany).

Sequence database and phylogenetic tree of *Fermentibacteria*

To obtain the most accurate and most current 16S rRNA gene based phylogenetic tree of the novel phylum *Fermentibacteria* (formerly candidate division Hyd24-12), we searched NCBI for related sequences using a Match/Mismatch Score (1, -1) allowing for a broad search, added the ~500 retrieved sequences to the non-redundant SILVA small subunit reference database (release 123.1; March 2016) (Quast *et al.*, 2013) using ARB (Ludwig *et al.*, 2004) and discarded all sequences that did not affiliate to the target clade in the SILVA reference tree. Additionally, we included 16S rRNA gene sequences of unpublished gene libraries obtained from methane seep ecosystems. These sequences were screened for chimeras using Mallard v1.02 (Ashelford *et al.*, 2006). Sequences were aligned using SINA (Pruesse *et al.*, 2012) and the alignment was manually optimized according to the rRNA secondary structure. We obtained 328 high-quality non-redundant sequences affiliating with *Fermentibacteria*, of which 255 were nearly full length (>1340 nucleotides). For non-redundancy we removed sequences that were >99% similar and originated from the same library. That means we kept sequences that were <99% similar and came from the same library, as well as sequences that were >99% similar, but from different libraries. *Fermentibacteria* sequences, alignments, and phylogenetic trees are provided as ARB database (Dataset 3).

Phylogenomic tree reconstruction

Phylogenomic affiliation of bin O was calculated based on 37 bacterial single copy genes. We used Phylosift (v1.0.1) (Darling *et al.*, 2014) to retrieve and align the 37 single copy marker genes from bin O and from three previously published provisional genomes affiliated with *Fermentibacteria* (Kirkegaard *et al.*, 2016). The concatenated marker alignment was merged with the reference alignment from the Phylosift database (v1413946442), which contains over 4000 additional genomes. The resulting multi-locus alignment was used to generate a phylogenomic tree using Fasttree (v2.1.2 SSE3 with OpenMP) (Price *et al.*, 2010) with increased rounds of minimum evolution SPR (-spr 4) and exhaustive maximum likelihood nearest neighbor interchanges (-mlacc 2 -slownni). Subsequently, the tree was optimized with ARB and the leaves were grouped according to their phylogenetic affiliation on phylum level.

**Supporting Results**

Global occurrence of *Fermentibacteria* in anoxic, organic-rich ecosystems

The phylum-level clade *Fermentibacteria* (formerly candidate division Hyd24-12) contains almost exclusively sequences that were retrieved from anoxic, organic and/or methane-rich ecosystems, such as sulfidic cave biofilms (Macalady *et al.*, 2006), sulfur-rich springs (Elshahed *et al.*, 2007), methane seeps (Knittel *et al.*, 2003; Heijs *et al.*, 2008; Pernthaler *et al.*, 2008; Beal *et al.*, 2009; Lloyd *et al.*, 2010; Yanagawa *et al.*, 2011; Marlow *et al.*, 2014; Ruff *et al.*, 2015; Trembath-Reichert *et al.*, 2016), marine mud volcanoes (Niemann *et al.*, 2006; Omoregie *et al.*, 2008; Pachiadaki *et al.*, 2010, 2011), terrestrial mud volcanoes (Cheng *et al.*, 2012) methane hydrates (Mills *et al.*, 2005), AOM enrichment cultures (Schreiber *et al.*, 2010; Aoki *et al.*, 2014), hydrothermal sediments (McKay *et al.*, 2016), sulfur-rich marine sediments (Schauer *et al.*, 2011), coral reef sands (Schöttner *et al.*, 2011) shelf sediments (Köchling *et al.*, 2011; Julies *et al.*, 2012), sunken wood (Fagervold *et al.*, 2012), anoxic hypersaline microbial mats (Ley *et al.*, 2006; Arp *et al.*, 2012; Harris *et al.*, 2013; Schneider *et al.*, 2013), in marine sponges (Simister *et al.*, 2012), anaerobic digesters (Hatamoto *et al.*, 2007; Satoh *et al.*, 2007; Arnett *et al.*, 2009; Xing *et al.*, 2010; Nelson *et al.*, 2012; Kirkegaard *et al.*, 2016) and landfill leachate (Liu *et al.*, 2011).

**Supporting Figures and Tables**

**Figure S1**


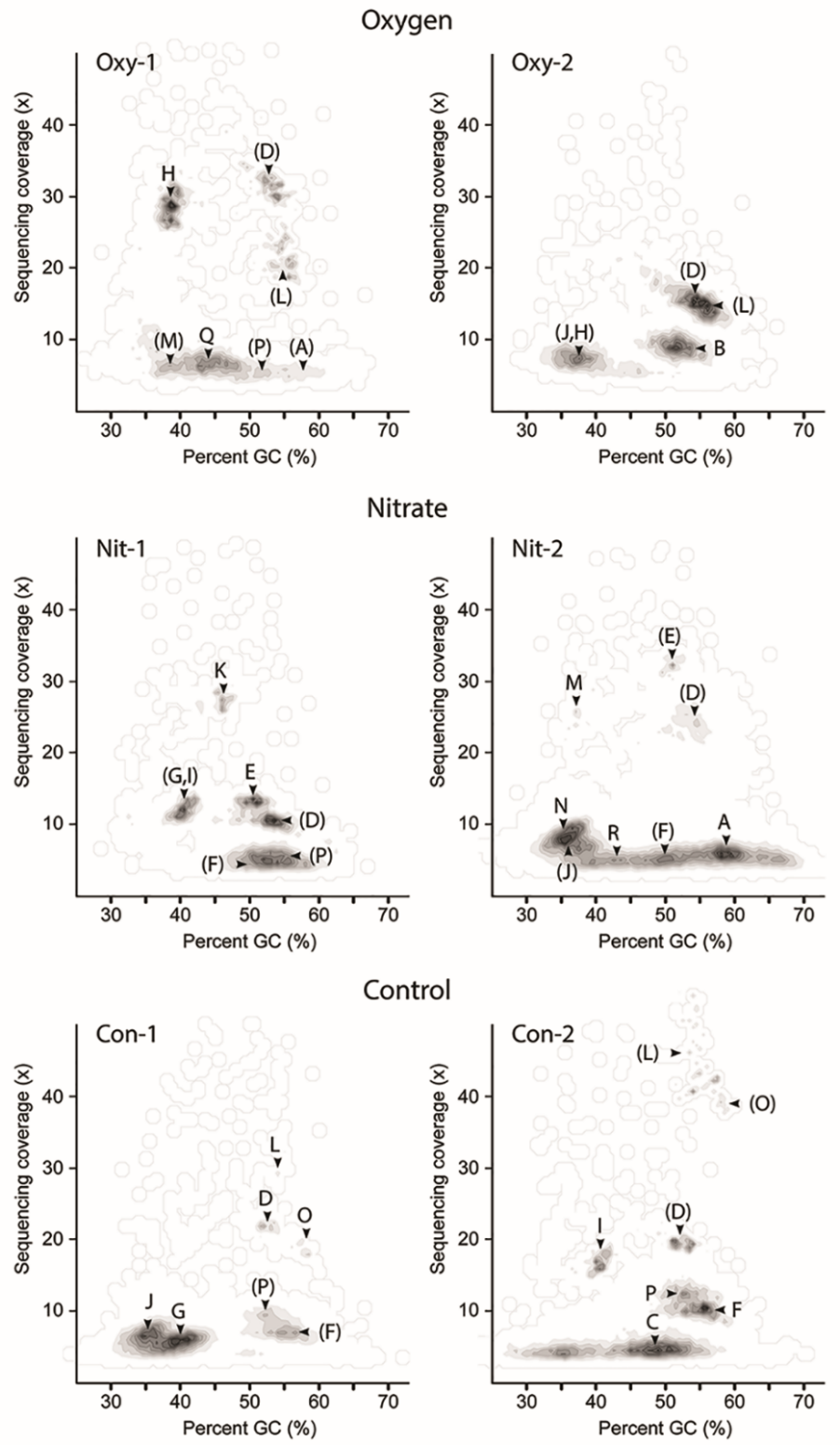


Figure S1. Visualization of assembled contigs of the six metagenomes (Oxy-1, Oxy-2, Nit-1, Nit-2, Con-1, Con-2) after binning. Bins A-R are indicated with capital letters. Letters in parentheses show large bins that were detected, but had assembled better in another sample. Refer to this graph to link the metatranscriptomic contig identifier (Dataset 1) to the metagenome that contains the respective bin/contig. Assembled contigs are archived in the SRA under: Oxy-1: LQAE00000000; Oxy-2:LQAF00000000; Nit-1: LQAG00000000; Nit-2: LQAH00000000; Con-1: LQAI00000000; Con-2: LQAJ00000000

**Figure S2**


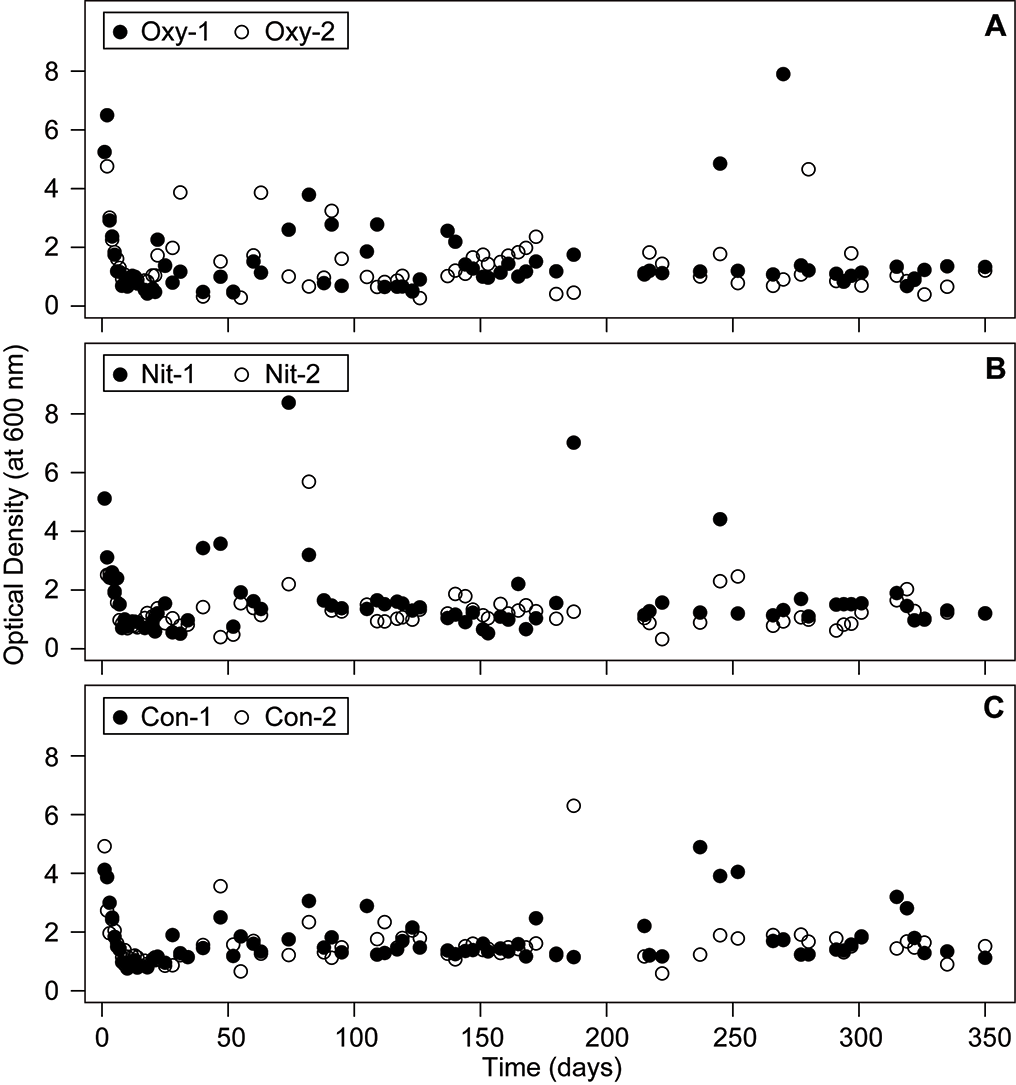


Figure S2: The optical density and dilution rate remained constant during the entire experiment. This means that microbial growth and biomass remained constant in each culture and were not affected by the different treatments. Based on the dilution rate /growth rate of 0.36 – 0.4, the organisms had an average doubling time of 40 - 45 hours. Optical density was measured as absorbance at 600 nm wavelength (OD_600_).

**Figure S3**


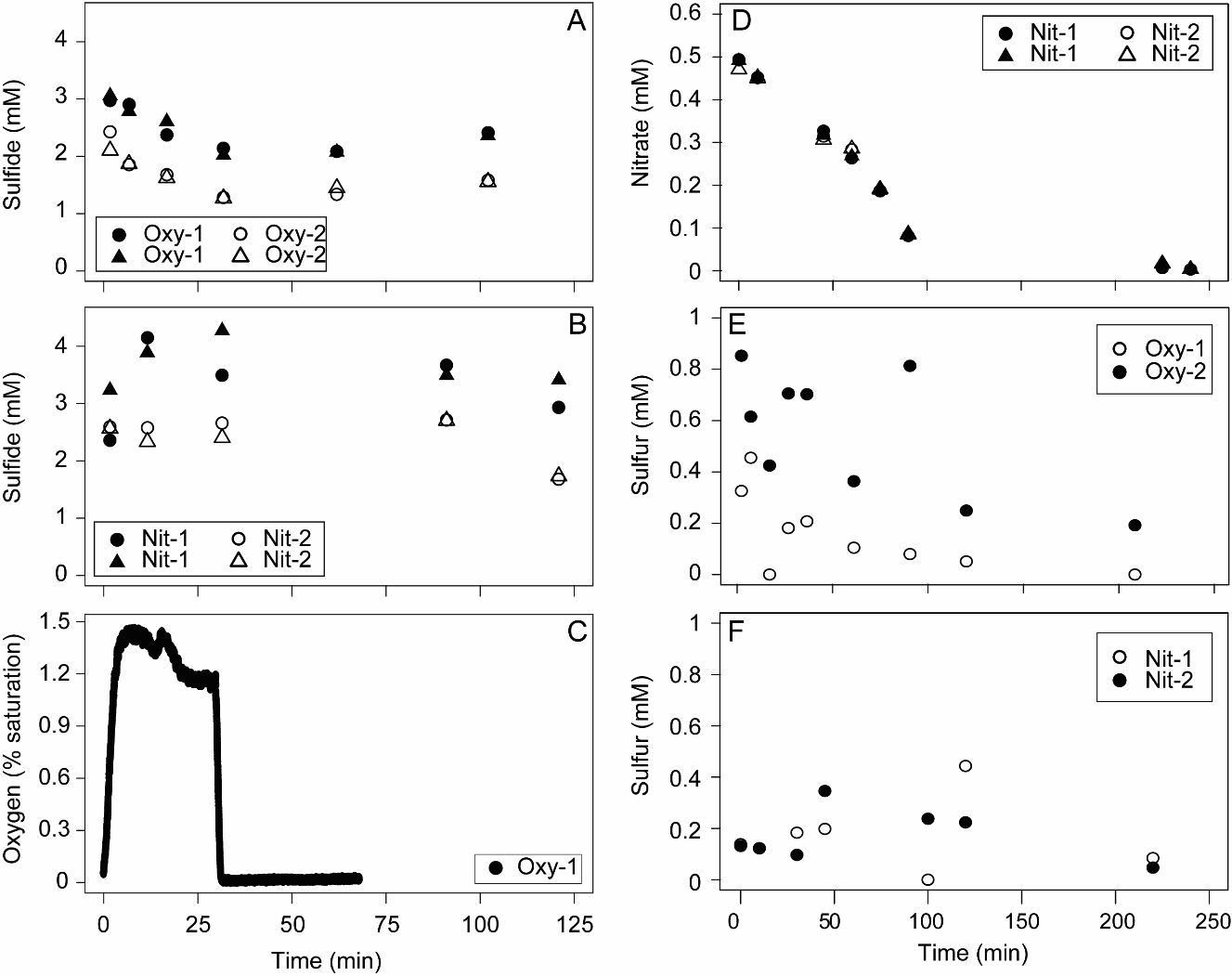


Figure S3: The concentrations of sulfide, oxygen or nitrate during one treatment cycle. The sulfide concentration decreased during aeration and recovered when the aeration was terminated after 30 min (A). The sulfide concentration in the nitrate treatment (B) was fluctuating, but constant. Oxygen reached a concentration of 1.4% saturation during the treatment (C) and was flushed out of the culture after 30 min using argon. Nitrate reached 0.5 mM during the treatment and was metabolized by the microorganisms within 100 min (D). The sulfur concentration in the oxygen treated cultures was high immediately after the aeration and then decreased (E), whereas in the nitrate-treated cultures seemed to peak at around 100 minutes after the treatment.

**Figure S4**


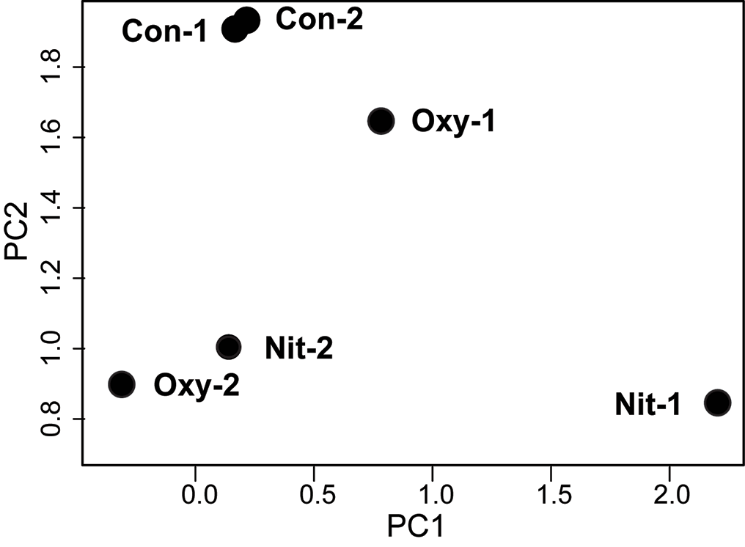


Figure S4: Principal component analysis (Edge-PCA) of the six Janssand continuous culture metagenomes based on relative abundance of 37 core marker gene sequences as detected by Phylosift.

**Figure S5**

**
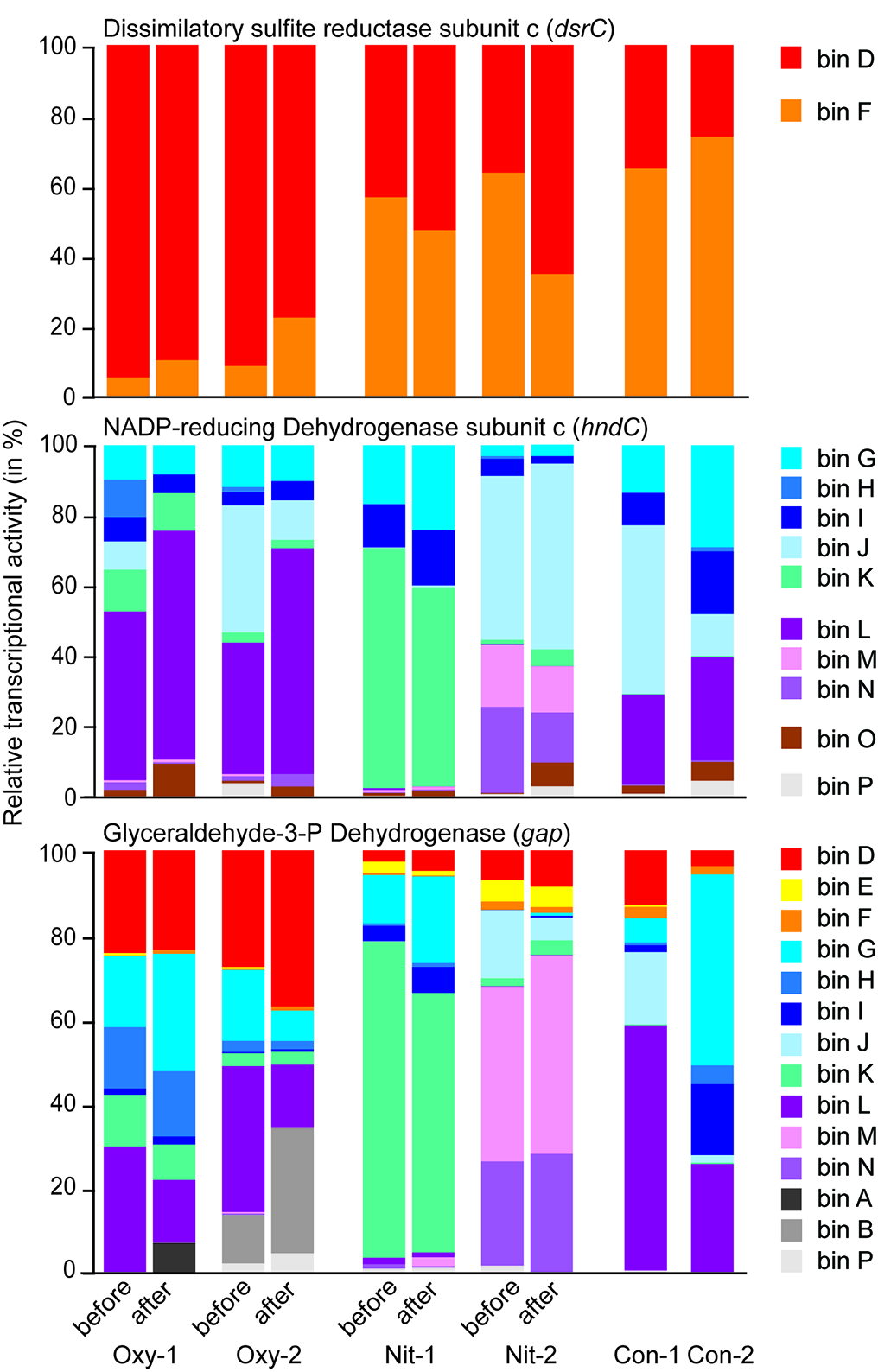
**

Figure S5. Relative transcriptional activity for key genes involved in sulfate reduction (*dsrC*), fermentation (*hndC*) and glycolysis (*gap*). The presented percentages of relative transcriptional activity were normalized for each bin and standardized using each bins relative abundance. This way it is possible to compare the relative transcriptional activity across bins and conditions.

**Figure S6**


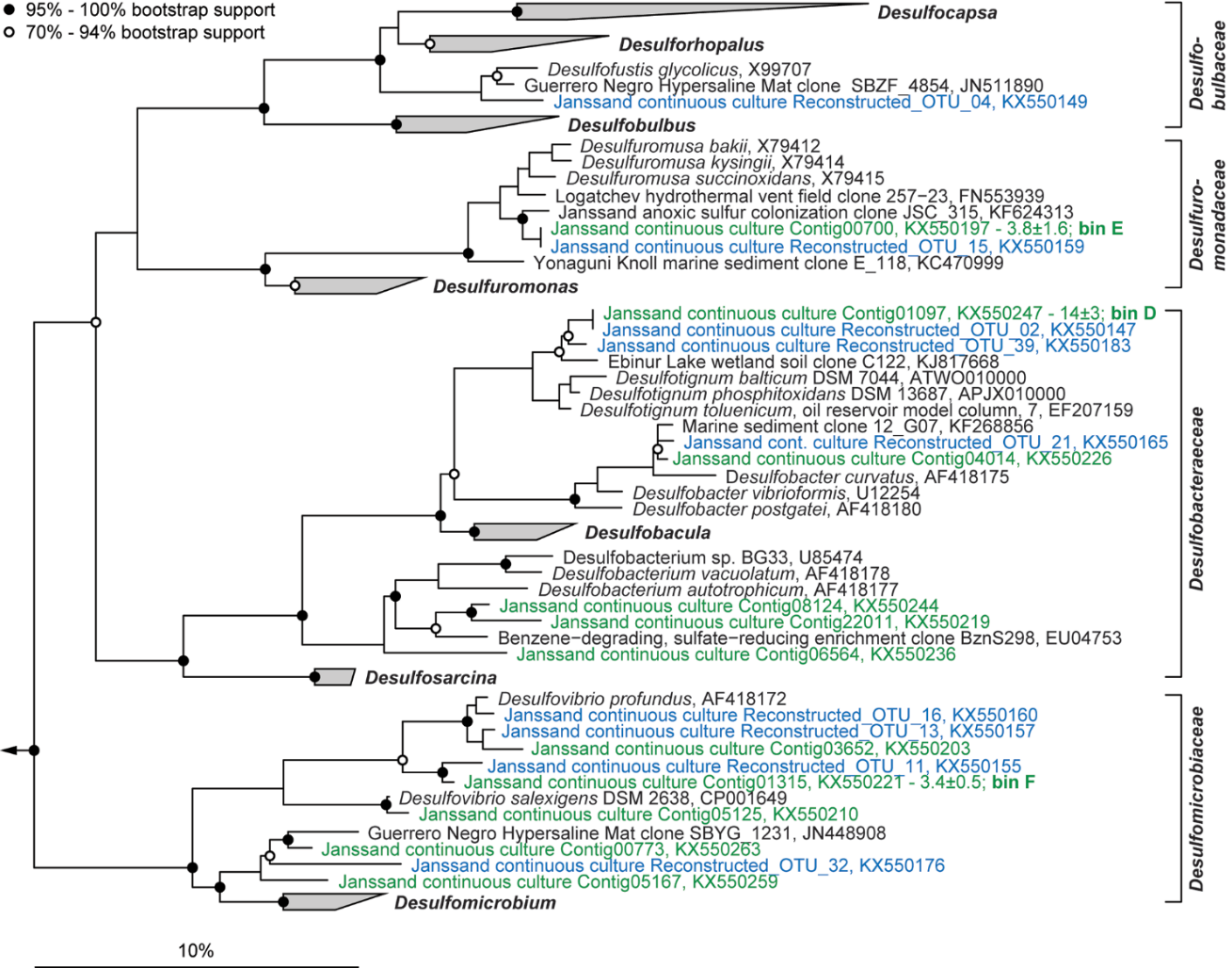


Figure S6: Phylogeny of 16S rRNA gene sequences that affiliated with the class *Deltaproteobacteria*. Sequences were reconstructed using Emirge (blue) or directly retrieved from contigs (green) of Janssand continuous culture metagenomes. Corresponding bins are indicated, including source contigs and average coverage (± S.D.) over all samples (in %). The phylogeny was calculated using the non-redundant SILVA small subunit reference database (v123.1, release 03/2016) and phyml maximum likelihood with 100 iterations. Scale bar shows estimated sequence divergence.

**Figure S7**


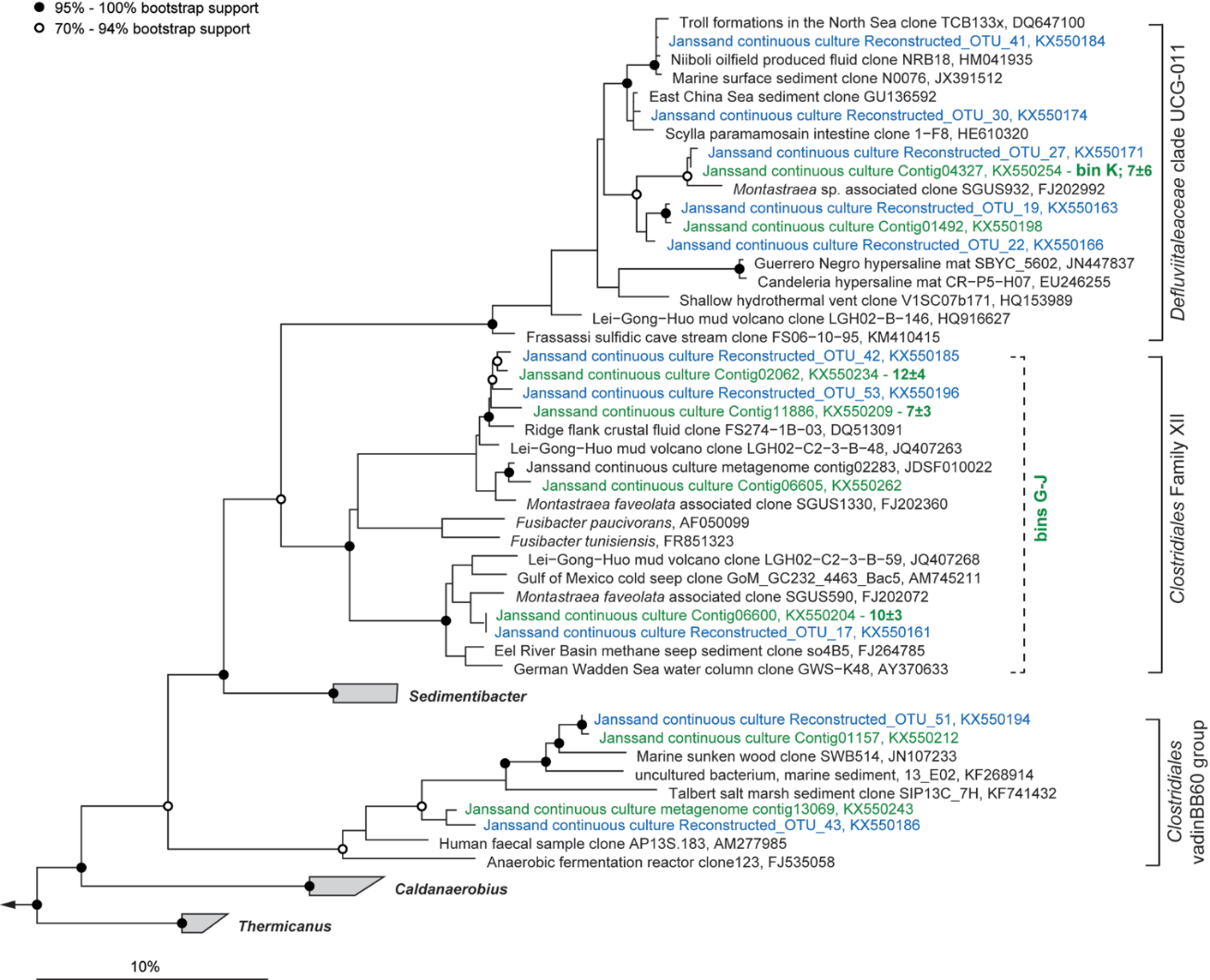


Figure S7. Phylogeny of 16S rRNA gene sequences that affiliated with the phylum *Clostridiales*. Sequences were reconstructed using Emirge (blue) or directly retrieved from contigs (green) of Janssand continuous culture metagenomes. Corresponding bins are indicated, including source contigs and average coverage (± S.D.) over all samples (in %). The phylogeny was calculated using the non-redundant SILVA small subunit reference database (v123.1, release 03/2016) and phyml maximum likelihood with 100 iterations. Scale bar shows estimated sequence divergence.

**Figure S8**


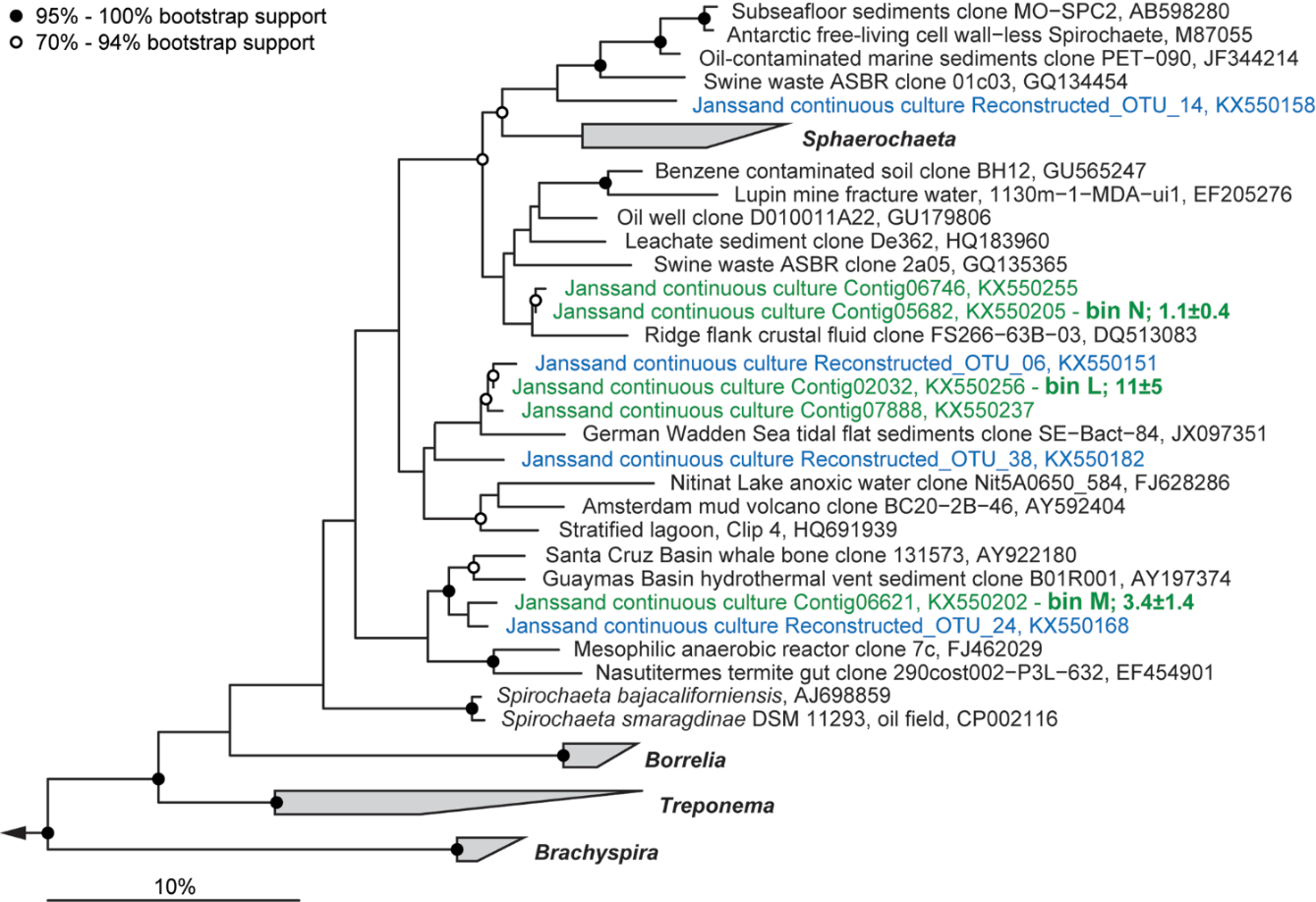


Figure S8. Phylogeny of 16S rRNA gene sequences that affiliated with the family *Spirochaetaceae* in the phylum *Spirochaeta*. Sequences were reconstructed using Emirge (blue) or directly retrieved from contigs (green) of Janssand continuous culture metagenomes. Corresponding bins are indicated, including source contigs and average coverage (± S.D.) over all samples (in %). The phylogeny was calculated using the non-redundant SILVA small subunit reference database (v123.1, release 03/2016) and phyml maximum likelihood with 100 iterations. Scale bar shows estimated sequence divergence.

**Figure S9**


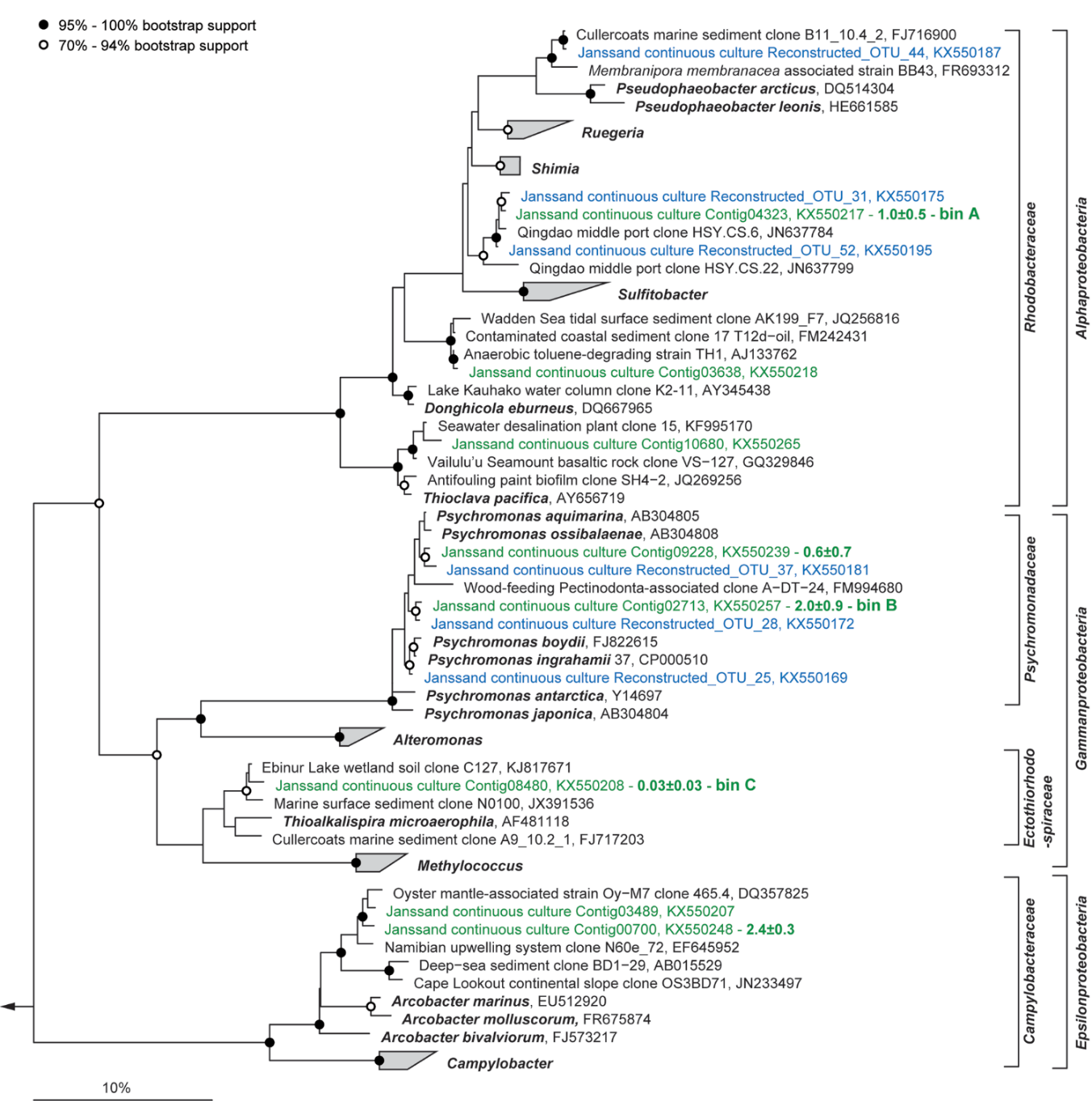


Figure S9. Phylogeny of 16S rRNA gene sequences that affiliated with *Alpha-, Gamma- and Epsilonproteobacteria*. Sequences were reconstructed using Emirge (blue) or directly retrieved from contigs (green) of Janssand continuous culture metagenomes. Corresponding bins are indicated, including source contigs and average coverage (± S.D.) over all samples (in %). The phylogeny was calculated using the non-redundant SILVA small subunit reference database (v123.1, release 03/2016) and phyml maximum likelihood with 100 iterations. Scale bar shows estimated sequence divergence.

**Figure S10**


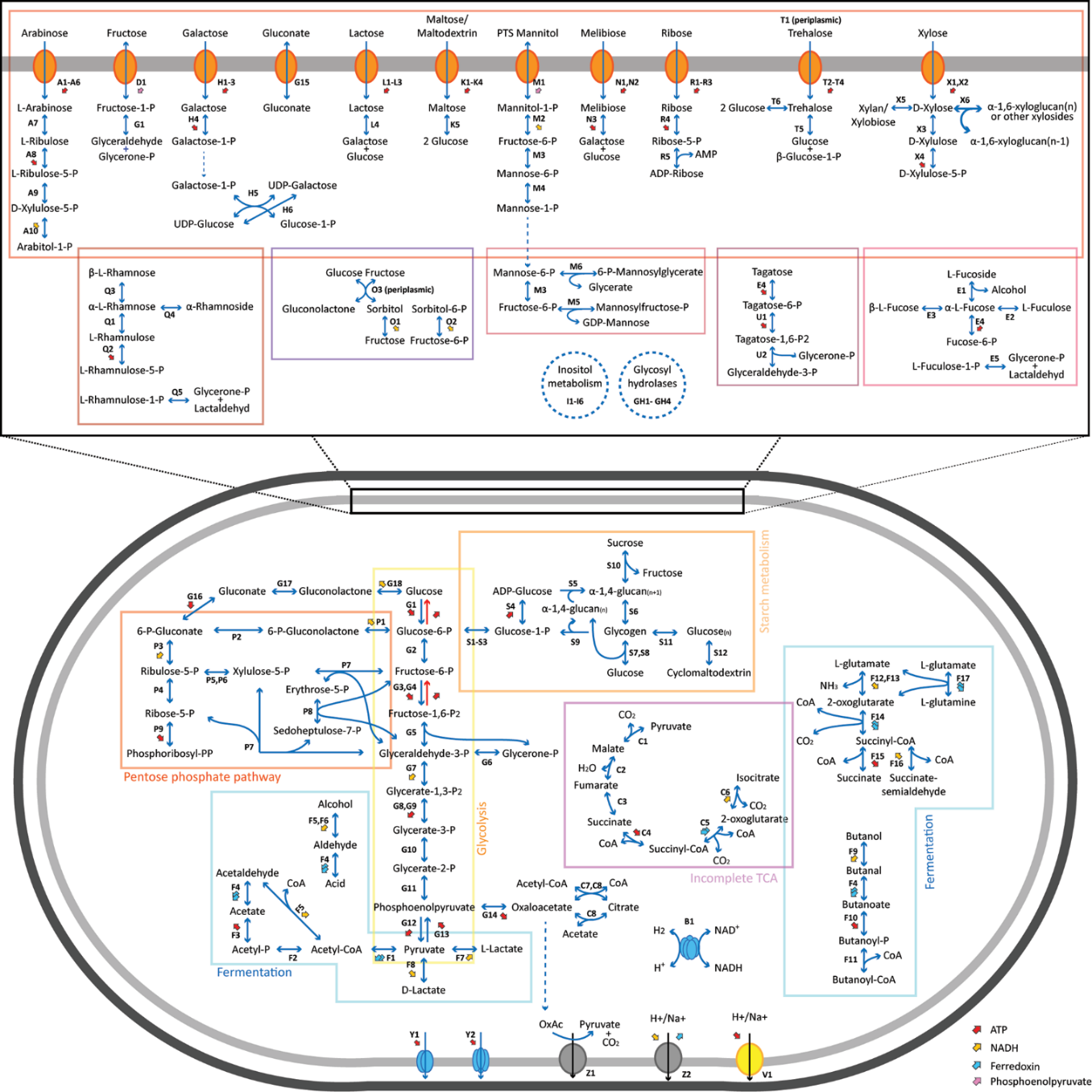


Figure S10: Metabolic map of *Spirochaeta*-affiliated bin L. Detected enzymes are shown as blue arrows, undetected enzymes as red arrows. Enzymes are abbreviated with letters, a full list as well as further metabolic pathways is provided in Table S3.

**Figure S11**


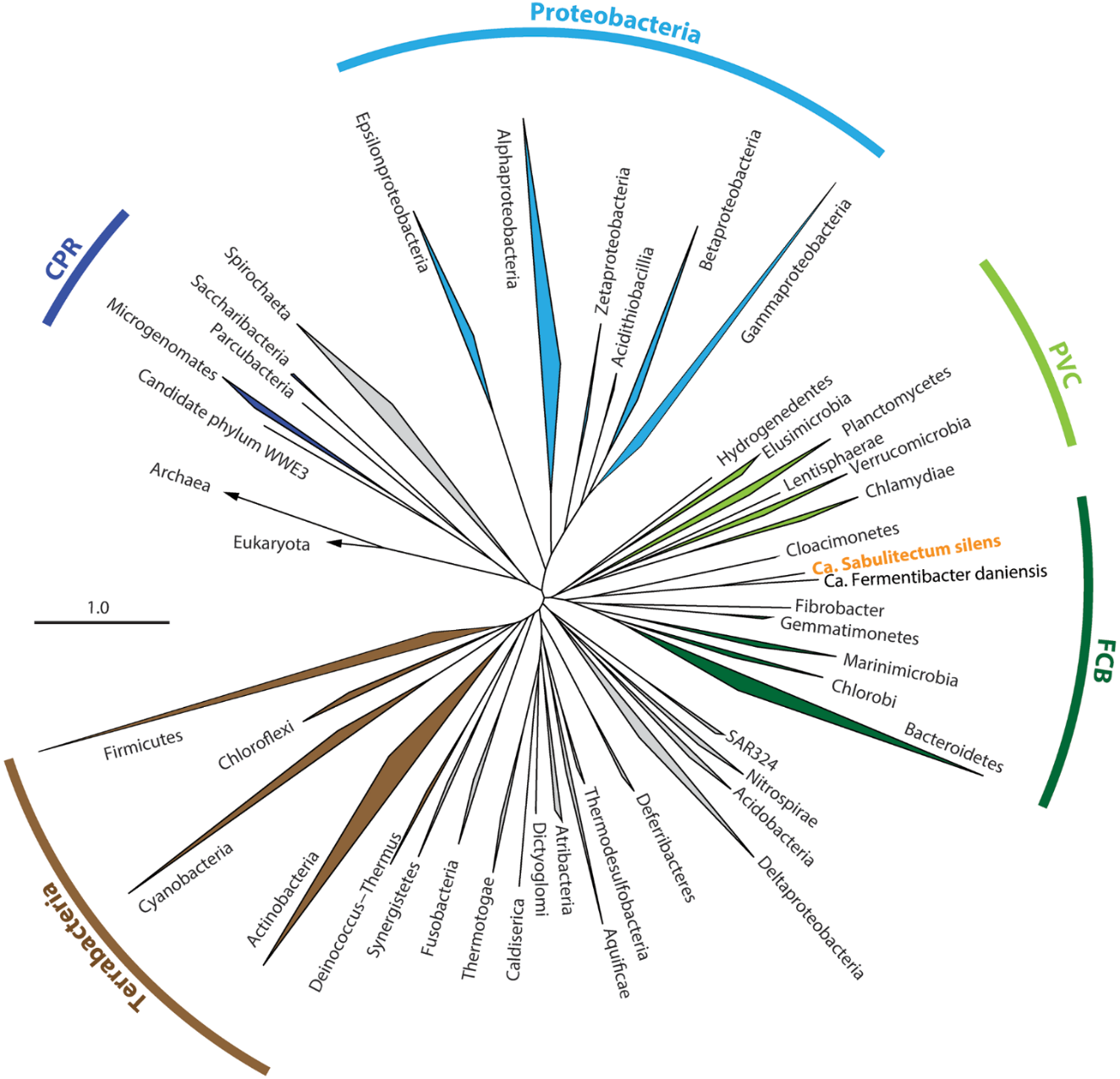


Figure S11. Phylogenomic placement of *^U^Sabulitectum silens* (bin O) and *Ca*. Fermentibacter daniensis based on a concatenated alignment of 37 bacterial single copy genes. Both provisional species belong to the *Fermentibacteria* within the superphylum FCB (*Fibrobacteres*, *Chlorobi*, *Bacteroidetes*). Note: The branch-lengths of *Gammaproteobacteria* and *Firmicutes* were shortened for better visualization.

**Table S1**

Table S1: Transcription (marked with X) of glycosyl hydrolase families in each bin (A-R)

|  | A | B | C | D | E | F | G | H | I | J | K | L | M | N | O | P | Q | | R |
| --- | --- | --- | --- | --- | --- | --- | --- | --- | --- | --- | --- | --- | --- | --- | --- | --- | --- | --- | --- |
| GH2 |  |  |  |  |  |  |  |  |  |  |  | X |  |  |  |  | |  |  |
| GH9 |  |  |  |  |  |  |  |  |  |  |  |  | X |  |  |  | |  |  |
| GH17 |  |  |  |  |  |  |  |  |  |  | X |  |  |  |  |  | |  |  |
| GH20 |  |  |  |  |  |  |  |  |  |  |  | X | X |  |  |  | |  |  |
| GH25 |  |  |  |  |  |  |  |  |  |  |  |  |  |  |  | X | |  |  |
| GH26 |  |  |  |  |  |  |  |  | X |  |  |  |  |  |  |  | |  |  |
| GH43 |  |  |  |  |  |  |  |  |  |  | X |  |  |  |  |  | |  |  |
| GH57 |  |  |  |  | X |  |  |  |  |  |  | X | X |  |  | X | |  | X |
| GH85 |  |  |  |  |  |  |  |  |  |  |  |  |  |  | X |  | |  |  |
| GH94 |  |  |  |  |  |  |  |  |  |  |  |  |  |  | X |  | |  |  |

**Table S2**

Table S2: Genome specifications of *^U^Sabulitectum silens* (JCC-6)*

| No. of contigs | 69 |
| --- | --- |
| Longest contig | 537883 |
| Total length of contigs | 2912633 |
| N50 | 221906 |
| N75 | 98298 |
| GC (%) | 56.6 |
| Genome completeness (%) | 76.9 |
| Genome contamination (%) | 5 |
| No. of conserved single copy genes | 98 |
| No. of tRNAs | 39 |
| rRNAs present | 5S, 16S, 23S |
| L50 | 4 |
| L75 | 9 |
| No. Of N's per 100 kbp | 0.00 |
|  |  |
| * based on contigs of >500bp |  |

**Supplementing References**

Aoki, M., Ehara, M., Saito, Y., Yoshioka, H., Miyazaki, M., Saito, Y., et al. (2014) A Long-Term Cultivation of an Anaerobic Methane-Oxidizing Microbial Community from Deep-Sea Methane-Seep Sediment Using a Continuous-Flow Bioreactor. *PLoS One* **9**: e105356.

Arnett, C.M., Rodriguez, G., and Maloney, S.W. (2009) Analysis of Bacterial Community Diversity in Anaerobic Fluidized Bed Bioreactors Treating 2,4-Dinitroanisole (DNAN) and n-Methyl-4-nitroaniline (MNA) Using 16S rRNA Gene Clone Libraries. *Microbes Environ.* **24**: 72–75.

Arp, G., Helms, G., Karlinska, K., Schumann, G., Reimer, A., Reitner, J., and Trichet, J. (2012) Photosynthesis versus Exopolymer Degradation in the Formation of Microbialites on the Atoll of Kiritimati, Republic of Kiribati, Central Pacific. *Geomicrobiol. J.* **29**: 29–65.

Ashelford, K.E., Chuzhanova, N.A., Fry, J.C., Jones, A.J., and Weightman, A.J. (2006) New Screening Software Shows that Most Recent Large 16S rRNA Gene Clone Libraries Contain Chimeras. *Appl. Environ. Microbiol.* **72**: 5734–5741.

Beal, E.J., House, C.H., and Orphan, V.J. (2009) Manganese- and iron-dependent marine methane oxidation. *Science* **325**: 184–187.

Cheng, T.-W., Chang, Y.-H., Tang, S.-L., Tseng, C.-H., Chiang, P.-W., Chang, K.-T., et al. (2012) Metabolic stratification driven by surface and subsurface interactions in a terrestrial mud volcano. *ISME J.* **6**: 2280–2290.

Darling, A.E., Jospin, G., Lowe, E., Matsen, F.A.I. V, Bik, H.M., and Eisen, J.A. (2014) PhyloSift: phylogenetic analysis of genomes and metagenomes. *PeerJ* **2**: e243.

Elshahed, M.S., Youssef, N.H., Luo, Q., Najar, F.Z., Roe, B.A., Sisk, T.M., et al. (2007) Phylogenetic and Metabolic Diversity of Planctomycetes from Anaerobic, Sulfide- and Sulfur-Rich Zodletone Spring, Oklahoma. *Appl. Environ. Microbiol.* **73**: 4707–4716.

Fagervold, S.K., Galand, P.E., Zbinden, M., Gaill, F., Lebaron, P., and Palacios, C. (2012) Sunken woods on the ocean floor provide diverse specialized habitats for microorganisms. *FEMS Microbiol. Ecol.* **82**: 616–628.

Hanke, A., Berg, J., Hargesheimer, T., Tegetmeyer, H.E., Sharp, C.E., and Strous, M. (2016) Selective Pressure of Temperature on Competition and Cross-Feeding within Denitrifying and Fermentative Microbial Communities. *Front. Microbiol.* **6**: 1461.

Harris, J.K., Caporaso, J.G., Walker, J.J., Spear, J.R., Gold, N.J., Robertson, C.E., et al. (2013) Phylogenetic stratigraphy in the Guerrero Negro hypersaline microbial mat. *ISME J.* **7**: 50–60.

Hatamoto, M., Imachi, H., Yashiro, Y., Ohashi, A., and Harada, H. (2007) Diversity of Anaerobic Microorganisms Involved in Long-Chain Fatty Acid Degradation in Methanogenic Sludges as Revealed by RNA-Based Stable Isotope Probing. *Appl. Environ. Microbiol.* **73**: 4119–4127.

Heijs, S.K., Laverman, A.M., Forney, L.J., Hardoim, P.R., and Van Elsas, J.D. (2008) Comparison of deep-sea sediment microbial communities in the Eastern Mediterranean. *FEMS Microbiol. Ecol.* **64**: 362–377.

Julies, E.M., Brüchert, V., and Fuchs, B.M. (2012) Vertical shifts in the microbial community structure of organic-rich Namibian shelf sediments. *African J. Microbiol. Res.* **6**: 3887–3897.

Kirkegaard, R.H., Dueholm, M.S., McIlroy, S.J., Nierychlo, M., Karst, S.M., Albertsen, M., and Nielsen, P.H. (2016) Genomic insights into members of the candidate phylum Hyd24-12 common in mesophilic anaerobic digesters. *ISME J.* 1–13.

Knittel, K., Boetius, A., Lemke, A., Eilers, H., Lochte, K., Pfannkuche, O., et al. (2003) Activity, Distribution, and Diversity of Sulfate Reducers and Other Bacteria in Sediments above Gas Hydrate (Cascadia Margin, Oregon). *Geomicrobiol. J.* **20**: 269–294.

Köchling, T., Lara-Martín, P., González-Mazo, E., Amils, R., and Sanz, J.L. (2011) Microbial community composition of anoxic marine sediments in the Bay of Cádiz (Spain). *Int. Microbiol.* **14**: 143–154.

Ley, R.E., Harris, J.K., Wilcox, J., Spear, J.R., Miller, S.R., Bebout, B.M., et al. (2006) Unexpected diversity and complexity of the Guerrero Negro hypersaline microbial mat. *Appl. Environ. Microbiol.* **72**: 3685–3695.

Liu, J., Wu, W., Chen, C., Sun, F., and Chen, Y. (2011) Prokaryotic diversity, composition structure, and phylogenetic analysis of microbial communities in leachate sediment ecosystems. *Appl. Microbiol. Biotechnol.* **91**: 1659–1675.

Lloyd, K.G., Albert, D.B., Biddle, J.F., Chanton, J.P., Pizarro, O., and Teske, A. (2010) Spatial Structure and Activity of Sedimentary Microbial Communities Underlying a Beggiatoa spp. Mat in a Gulf of Mexico Hydrocarbon Seep. *PLoS One* **5**: e8738.

Ludwig, W., Strunk, O., Westram, R., Richter, L., Meier, H., Yadhukumar, et al. (2004) ARB: a software environment for sequence data. *Nucleic Acids Res.* **32**: 1363–1371.

Macalady, J.L., Lyon, E.H., Koffman, B., Albertson, L.K., Meyer, K., Galdenzi, S., and Mariani, S. (2006) Dominant Microbial Populations in Limestone-Corroding Stream Biofilms, Frasassi Cave System, Italy. *Appl. Environ. Microbiol.* **72**: 5596–5609.

Marlow, J.J., Steele, J. a., Case, D.H., Connon, S. a., Levin, L. a., and Orphan, V.J. (2014) Microbial abundance and diversity patterns associated with sediments and carbonates from the methane seep environments of Hydrate Ridge, OR. *Front. Mar. Sci.* **1**: 1–16.

McKay, L., Klokman, V.W., Mendlovitz, H.P., LaRowe, D.E., Hoer, D.R., Albert, D., et al. (2016) Thermal and geochemical influences on microbial biogeography in the hydrothermal sediments of Guaymas Basin, Gulf of California. *Environ. Microbiol. Rep.* **8**: 150–161.

Mills, H.J., Martinez, R.J., Story, S., and Sobecky, P.A. (2005) Characterization of Microbial Community Structure in Gulf of Mexico Gas Hydrates: Comparative Analysis of DNA- and RNA-Derived Clone Libraries. *Appl. Environ. Microbiol.* **71**: 3235–3247.

Nelson, M.C., Morrison, M., Schanbacher, F., and Yu, Z. (2012) Shifts in microbial community structure of granular and liquid biomass in response to changes to infeed and digester design in anaerobic digesters receiving food-processing wastes. *Bioresour. Technol.* **107**: 135–143.

Niemann, H., Duarte, J., Hensen, C., Omoregie, E., Magalhàes, V.H., Elvert, M., et al. (2006) Microbial methane turnover at mud volcanoes of the Gulf of Cadiz. *Geochim. Cosmochim. Acta* **70**: 5336–5355.

Omoregie, E.O., Mastalerz, V., de Lange, G., Straub, K.L., Kappler, A., Røy, H., et al. (2008) Biogeochemistry and Community Composition of Iron- and Sulfur-Precipitating Microbial Mats at the Chefren Mud Volcano (Nile Deep Sea Fan, Eastern Mediterranean). *Appl. Environ. Microbiol.* **74**: 3198–3215.

Pachiadaki, M.G., Kallionaki, A., Dählmann, A., De Lange, G.J., and Kormas, K.A. (2011) Diversity and Spatial Distribution of Prokaryotic Communities Along A Sediment Vertical Profile of A Deep-Sea Mud Volcano. *Microb. Ecol.* **62**: 655–668.

Pachiadaki, M.G., Lykousis, V., Stefanou, E.G., and Kormas, K.A. (2010) Prokaryotic community structure and diversity in the sediments of an active submarine mud volcano (Kazan mud volcano, East Mediterranean Sea). *FEMS Microbiol. Ecol.* **72**: 429–444.

Pernthaler, A., Dekas, A.E., Brown, C.T., Goffredi, S.K., Embaye, T., and Orphan, V.J. (2008) Diverse syntrophic partnerships from deep-sea methane vents revealed by direct cell capture and metagenomics. *Proc. Natl. Acad. Sci. U. S. A.* **105**: 7052–7.

Price, M.N., Dehal, P.S., and Arkin, A.P. (2010) FastTree 2 – Approximately Maximum-Likelihood Trees for Large Alignments. *PLoS One* **5**: e9490.

Pruesse, E., Peplies, J., and Glockner, F.O. (2012) SINA: Accurate high-throughput multiple sequence alignment of ribosomal RNA genes. *Bioinformatics* **28**: 1823–1829.

Quast, C., Pruesse, E., Yilmaz, P., Gerken, J., Schweer, T., Yarza, P., et al. (2013) The SILVA ribosomal RNA gene database project: improved data processing and web-based tools. *Nucleic Acids Res.* **41**: D590–D596.

Ruff, S.E., Biddle, J.F., Teske, A.P., Knittel, K., Boetius, A., and Ramette, A. (2015) Global dispersion and local diversification of the methane seep microbiome. *Proc. Natl. Acad. Sci. U. S. A.* **112**: 4015–20.

Satoh, H., Miura, Y., Tsushima, I., and Okabe, S. (2007) Layered Structure of Bacterial and Archaeal Communities and Their In Situ Activities in Anaerobic Granules. *Appl. Environ. Microbiol.* **73**: 7300–7307.

Schauer, R., Røy, H., Augustin, N., Gennerich, H.-H., Peters, M., Wenzhoefer, F., et al. (2011) Bacterial sulfur cycling shapes microbial communities in surface sediments of an ultramafic hydrothermal vent field. *Environ. Microbiol.* **13**: no-no.

Schneider, D., Arp, G., Reimer, A., Reitner, J., and Daniel, R. (2013) Phylogenetic Analysis of a Microbialite-Forming Microbial Mat from a Hypersaline Lake of the Kiritimati Atoll, Central Pacific. *PLoS One* **8**: e66662.

Schöttner, S., Pfitzner, B., Grünke, S., Rasheed, M., Wild, C., and Ramette, A. (2011) Drivers of bacterial diversity dynamics in permeable carbonate and silicate coral reef sands from the Red Sea. *Environ. Microbiol.* **13**: 1815–1826.

Schreiber, L., Holler, T., Knittel, K., Meyerdierks, A., and Amann, R. (2010) Identification of the dominant sulfate-reducing bacterial partner of anaerobic methanotrophs of the ANME-2 clade. *Environ. Microbiol.* **12**: 2327–2340.

Simister, R.L., Deines, P., Botté, E.S., Webster, N.S., and Taylor, M.W. (2012) Sponge-specific clusters revisited: a comprehensive phylogeny of sponge-associated microorganisms. *Environ. Microbiol.* **14**: 517–524.

Trembath-Reichert, E., Case, D.H., and Orphan, V.J. (2016) Characterization of microbial associations with methanotrophic archaea and sulfate-reducing bacteria through statistical comparison of nested Magneto-FISH enrichments. *PeerJ* **4**: e1913.

Xing, W., Zhao, Y., and Zuo, J. -e. (2010) Microbial activity and community structure in a lake sediment used for psychrophilic anaerobic wastewater treatment. *J. Appl. Microbiol.* **109**: 1829–1837.

Yanagawa, K., Sunamura, M., Lever, M.A., Morono, Y., Hiruta, A., Ishizaki, O., et al. (2011) Niche Separation of Methanotrophic Archaea (ANME-1 and -2) in Methane-Seep Sediments of the Eastern Japan Sea Offshore Joetsu. *Geomicrobiol. J.* **28**: 118–129.
